# Supplementary material for: Translation, validation and cultural adaptation of the Arabic version of the HIV knowledge questionnaire (HIV-Kq-18)
Source: PLoS One. 2023 Apr 13;18(4):e0284542. doi: 10.1371/journal.pone.0284542 (PMC10101484; doi:10.1371/journal.pone.0284542)
Supplement: S1 Appendix — (DOCX) [file pone.0284542.s001.docx]

**Supplementary appendix 1**: Arabic version of the HIV knowledge questionnaire (HIV-Kq-18)

**" استبيان قياس مدى المعرفة تجاه فيروس نقص المناعة البشري – ١٨** **"**

**فيما يلي مجموعة من الأسئلة يرجى اختيار خيار واحد فقط (من فضلك لا تقم بتخمين الإجابات في حاله عدم معرفه الإجابة اختر لا أعلم)**

| **لا أعلم** | **خطأ** | **صح** | **السؤال** |
| --- | --- | --- | --- |
|  |  |  | 1. عدوى فيروس نقص المناعة البشرية لا تنتقل عبر العطس أو السعال. |
|  |  |  | 1. يمكن أن يصاب الشخص بعدوى فيروس نقص المناعة البشرية عند الشرب من نفس الكوب الذي شرب منه شخص مصاب بالفيروس. |
|  |  |  | 1. أثناء الجماع، سحب قضيب الرجل قبل القذف يحمي المرأة من الإصابة بعدوى فيروس نقص المناعة البشرية. |
|  |  |  | 1. يمكن أن تصاب المرأة بفيروس نقص المناعة البشرية إذا مارست مع رجل الجماع الشرجي. |
|  |  |  | 1. الاستحمام أو غسل الأعضاء التناسلية بعد الجماع يحمي الشخص من الإصابة بفيروس نقص المناعة البشرية. |
|  |  |  | 1. جميع النساء الحوامل المصابات بفيروس نقص المناعة البشرية سيلدون أطفالاً مصابين بمرض الإيدز. |
|  |  |  | 1. تظهر على الأشخاص المصابين بفيروس نقص المناعة البشرية بسرعة علامات خطيرة للإصابة. |
|  |  |  | 1. هناك لقاح يحمي البالغين من الإصابة بفيروس نقص المناعة البشرية. |
|  |  |  | 1. من المرجح أن يصاب الأشخاص بفيروس نقص المناعة البشرية عن طريق التقبيل العميق، ووضع لسانهم في فم شريكهم، إذا كان شريكهم مصابًا بفيروس نقص المناعة البشرية. |
|  |  |  | 1. لا يمكن للمرأة الإصابة بفيروس نقص المناعة البشرية إن مارست الجماع خلال دورتها الشهرية. |
|  |  |  | 1. يوجد واقي أنثوي يمكنه تقليل فرص إصابة المرأة بعدوى فيروس نقص المناعة البشرية. |
|  |  |  | 1. الواقي الذكري المصنوع من الجلد الطبيعي أفضل في الحماية من فيروس الإيدز من الواقي المصنوع من المطاط (اللاتيكس). |
|  |  |  | 1. لن يصاب الشخص بفيروس نقص المناعة البشرية إن قام/قامت بأخذ المضادات الحيوية. |
|  |  |  | 1. ممارسة الجماع مع أكثر من شريك يمكنها زيادة فرص الإصابة بعدوى فيروس نقص المناعة البشرية. |
|  |  |  | 1. إجراء شخص اختبار فحص لفيروس نقص المناعة البشرية بعد أسبوع من ممارسة الجماع، يُمكنه من معرفة إذا كان مصاباً بالفيروس أم لا. |
|  |  |  | 1. يمكن للشخص ان يُصاب بعدوى فيروس نقص المناعة البشرية إن جلس في مسبح أو حوض استحمام ساخن مع شخص مصاب بالفيروس. |
|  |  |  | 1. يمكن الإصابة بعدوى فيروس نقص المناعة البشرية بواسطة ممارسة الجنس الفموي. |
|  |  |  | 1. استخدام الفازلين أو زيت الأطفال مع الواقي الذكري أثناء الجماع يقلل من فرص الإصابة بفيروس نقص المناعة البشرية. |

**ملحوظة هامة:** يرجى العلم أن فيروس نقص المناعة البشرية هو الفيروس المسبب لمتلازمة الإيدز.
