# Supplementary material for: Translation, validation and cultural adaptation of the Arabic version of the HIV knowledge questionnaire (HIV-Kq-18)
Source: PLoS One. 2023 Apr 13;18(4):e0284542. doi: 10.1371/journal.pone.0284542 (PMC10101484; doi:10.1371/journal.pone.0284542)
Supplement: Attachment — Submitted filename: PONE-D-23-01822-Re.pdf [file pone.0284542.s002.pdf]

Dear Editor-in-Chief

The manuscript ‘Validation and cultural adaptation of the Arabic version of the HIV knowledge questionnaire (HIV-Kq-18)′ addresses an interesting problem in the use of HIV-18 KQ. Although there are some points and concepts need to be fixed in order to be acceptable for publishing.

Here are some minor comments:

- Line 118 : “implying that a 10:1 ratio” must be changed by “implying that a 10.1 ratio”
- Line 174: “RStudio Version” must be changed by “R Studio Version”
- Line 174: “nFactors” must be changed by “n Factors”
- Line 175: “validateR” must be changed by “validate R”
- In page 19 the figure named “Adaptation and Validation of HIV-KQ-18 for Arabic Population”
- is Figure 1 not 3.
- In page 20 the figure named “The non-graphical solution of Scree plot to determine the number of factors to be retained” is Figure 2 not 4.
- In page 21 the table named “Participants’ characteristics” is Table 1 not 3.
- In page 18 the “**Table 2:** Factor analysis of HIV-KQ-18 Arabic Version” must be
  Table 4: Factor analysis of HIV-KQ-18 Arabic Version

Finally, after updating the manuscript upon these minor comments my opinion the manuscript can be accepted and published in your respective journal.

Best Regards
