# Supplementary material for: Translation, validation and cultural adaptation of the Arabic version of the HIV knowledge questionnaire (HIV-Kq-18)
Source: PLoS One. 2023 Apr 13;18(4):e0284542. doi: 10.1371/journal.pone.0284542 (PMC10101484; doi:10.1371/journal.pone.0284542)
Supplement: Attachment — Submitted filename: Point-by-point response to reviewers of round 1.docx [file pone.0284542.s003.docx]

**Point-by-point response**

**Manuscript Number:** PONE-D-23-01822

**Title of Article:** Translation, Validation and cultural adaptation of the Arabic version of the HIV knowledge questionnaire (HIV-Kq-18)

**Name of the Corresponding Author:** Elfatih A. Hasabo

**Email Address of the Corresponding Author:** [elfatih.ahmed.hasabo@gmail.com](mailto:elfatih.ahmed.hasabo@gmail.com)

**Journal Requirements:**

**Specific Responses:**

**1. Please ensure that your manuscript meets PLOS ONE's style requirements**

**Reply:**

**The manuscript now meets PLOS ONE's style requirements.**

**2. Please provide additional details regarding participant consent. In the ethics statement in the Methods and online submission information, please ensure that you have specified (1) whether consent was informed and**

**(2) what type you obtained (for instance, written or verbal, and if verbal, how it was documented and witnessed).**

**If your study included minors, state whether you obtained consent from parents or guardians. If the need for consent was waived by the ethics committee, please include this information.**

**Reply:**

**It is mentioned in detail in the ethical approval and consent to participate. And we clarified it more, thanks.**

**3. We note that you have indicated that data from this study are available upon request. PLOS only allows data to be available upon request if there are legal or ethical restrictions on sharing data publicly.**

**Reply:**

**We uploaded it.**

**In your revised cover letter, please address the following prompts:**

**a) If there are ethical or legal restrictions on sharing a de-identified data set, please explain them in detail (e.g., data contain potentially sensitive information, data are owned by a third-party organization, etc.) and who has imposed them (e.g., an ethics committee). Please also provide contact information for a data access committee, ethics committee, or other institutional body to which data requests may be sent.**

**b) If there are no restrictions, please upload the minimal anonymized data set necessary to replicate your study findings as either Supporting Information files or to a stable, public repository and provide us with the relevant URLs, DOIs, or accession numbers.**

**Reply:**

**We uploaded the data that you can use to replicate our study findings.**

**4. One of the noted authors is a group or consortium: Alpha Research Organization (ARO) team of collaborators**

**In addition to naming the author group, please list the individual authors and affiliations within this group in the acknowledgments section of your manuscript. Please also indicate clearly a lead author for this group along with a contact email address.**

**Reply: Added to the acknowledgement section with the affiliation. A contact email will be added to the submission system as a contact email. Please, we want to add the names of the collaborators mentioned in the acknowledgement to the ARO team of Collaborators when it is clicked in the author section.**

**5. Your ethics statement should only appear in the Methods section of your manuscript. If your ethics statement is written in any section besides the Methods, please move it to the Methods section and delete it from any other section. Please ensure that your ethics statement is included in your manuscript, as the ethics statement entered into the online submission form will not be published alongside your manuscript.**

**Reply: Moved to the methods section.**

**6. Please upload a copy of Figure 1 and 2, to which you refer in your text on page 8 and 10. If the figure is no longer to be included as part of the submission, please remove all reference to it within the text.**

**Reply: Figures 1 and 2 were uploaded in the text and we changed the mistake in numbering the figures.**

**7. Please ensure that you refer to Figure 3 and 4 in your text as, if accepted, production will need this reference to link the reader to the figure.**

**Reply: There are only figure 1 and 2 and we corrected them in the text. Figures 3 and 4 don’t exist we just number them incorrectly.**

**8. Please review your reference list to ensure that it is complete and correct. If you have cited papers that have been retracted, please include the rationale for doing so in the manuscript text or remove these references and replace them with relevant current references. Any changes to the reference list should be mentioned in the rebuttal letter that accompanies your revised manuscript. If you need to cite a retracted article, indicate the article’s retracted status in the References list and also include a citation and full reference for the retraction notice.**

**Reply:
We just added one reference (N.12) upon the request of reviewer 2.**

**Respond to reviewer 1**

We would like to thank reviewer 1 for all his effort during reviewing our manuscript.

**Specific Responses:**

***Comments:***

***• Line 118: “implying that a 10:1 ratio” must be changed by “implying that a 10.1 ratio”***

***• Line 174: “RStudio Version” must be changed by “R Studio Version”***

***• Line 174: “nFactors” must be changed by “n Factors”***

***• Line 175: “validateR” must be changed by “validate R”***

***• In page 19 the figure named “Adaptation and Validation of HIV-KQ-18 for Arabic Population” is Figure 1 not 3.***

***• In page 20 the figure named “The non-graphical solution of Scree plot to determine the number of factors to be retained” is Figure 2 not 4.***

***• In page 21 the table named “Participants’ characteristics” is Table 1 not 3.***

***• In page 18 the “Table 2: Factor analysis of HIV-KQ-18 Arabic Version” must be Table 4: Factor analysis of HIV-KQ-18 Arabic Version.”***

**Reply: The required amendments were done. Also, we uploaded Arabic version of the HIV knowledge questionnaire as a supplementary appendix.**

**Respond to reviewer 2**

We would like to thank reviewer 2 for all his effort during reviewing our manuscript.

**Specific Responses:**

***Comment 1:*** “

***• In the abstract and background on line 88 in the introduction, please provide the actual number of cases in addition to the percentage provided and also specify the time period.***

***• In the abstract, please, mention the validity metrics used to assess the questionnaire validity.***

***• Please, add reference for lines 121-122.***

***• A typo on line 132.”***

**Reply:**

- **We clarified this in the introduction section by adding a paragraph from line 87 – 94 and made it as requested.**
- **We mentioned the validity metrics from line 59 to 63 in the methods section of the abstract.**
- **We added a reference to lines 121 – 122.**
- **We rephrase the sentence on line 132.**
- **Also, we uploaded Arabic version of the HIV knowledge questionnaire as a supplementary appendix.**

Sincerely,

Elfatih A. Hasabo
